# Comparing the first and the second waves of COVID-19 in Italy: differences in epidemiological features and CT findings using a semi-quantitative score

**DOI:** 10.1007/s10140-021-01937-y

**Published:** 2021-07-29

**Authors:** Caterina Balacchi, Nicolò Brandi, Federica Ciccarese, Francesca Coppola, Vincenzo Lucidi, Laura Bartalena, Anna Parmeggiani, Alexandro Paccapelo, Rita Golfieri

**Affiliations:** Department of Radiology, IRCCS Azienda Ospedaliero-Universitaria Di Bologna, Via Albertoni 15, Bologna, Italia

**Keywords:** COVID-19, Coronavirus, SARS-CoV-2, CT, First wave, Second wave

## Abstract

**Purpose:**

CT findings of hospitalized COVID-19 patients were analyzed during both the first and the second waves of the pandemic, in order to detect any significant differences between the two groups.

**Methods:**

In this observational, retrospective, monocentric study, all hospitalized patients who underwent CT for suspected COVID-19 pneumonia from February 27 to March 27, 2020 (first wave) and from October 26 to November 24, 2020 (second wave) were enrolled. Epidemiological data, radiological pattern according to the RSNA consensus statement and visual score extension using a semi-quantitative score were compared.

**Results:**

Two hundred and eleven patients (mean age, 64.52 years ± 15.14, 144 males) were evaluated during the first wave while 455 patients (mean age, 68.26 years ± 16.34, 283 males) were studied during the second wave. The same prevalence of patterns was documented in both the first and the second waves (*p* = 0.916), with non-typical patterns always more frequently observed in elderly patients, especially the “indeterminate” pattern. Compared to those infected during the first wave, the patients of the second wave were older (64.52 vs.68.26, *p* = 0.005) and presented a slightly higher mean semi-quantitative score (9.0 ± 2.88 vs. 8.4 ± 3.06, *p* = 0.042). Age and semi-quantitative score showed a positive correlation (*r* = 0.15, *p* = 0.001).

**Conclusions:**

There was no difference regarding CT pattern prevalence between the first and the second waves, confirming both the validity of the RSNA consensus and the most frequent radiological COVID-19 features. Non-typical COVID-19 features were more frequently observed in older patients, thus should not be underestimated in the elderly population.

## Purpose

The ongoing COVID-19 pandemic has overwhelmed healthcare systems globally, imposing serious health, economic, and social effects on the population. Italy was the first European country to face the pandemic, having the first reported case detected on February 20, 2020, and it has been one of the most severely affected, with more than 250,000 COVID-19 cases until the end of August 2020 [1]. The risk of exceeding the capacity of the healthcare systems has led to strict lockdowns with universal movement restriction, involving social distancing measures. These measures have been effective in slowing down the pandemic, possibly reducing many millions of infections and averting the deaths of millions more; thus, they have been adopted as a model by many other countries. Moreover, several studies [2] have claimed that the seasonality of the immune response and the germicidal properties of solar UV rays during the summer months of 2020, as well as improved air quality due to city lockdowns [3], could have contributed to mitigating the COVID-19 epidemic, with a consequent reduction in both infection rate and criticality.

These hypotheses have been supported by a recent Italian study [4] in which it was demonstrated that the patients who died during this period (June–August 2020) were older, had more comorbidies and had a higher probability of superinfections compared to those infected during the first wave of the pandemic, suggesting that, during the summer, those most affected were the more fragile subject of the population.

However, as expected by many [2, 5], the easing of lockdowns and restrictions lead to a progressively and rapidly increase in new COVID-19 cases, causing a second wave of the pandemic with even higher numbers of victims and up to more than 40,000 new cases per day in November 2020 [1]. The continual increase in daily new COVID-19 cases was also accompanied also by an increase in the number of deaths, suggesting that the new wave did not reflect better diagnostic and therapeutic strategies [6].

At the end of March 2020, shortly after the dramatic worldwide spread of COVID-19, the Radiological Society of North America (RSNA) provided a consensus statement on reporting CT findings related to COVID-19 pneumonia, aimed to speed up and standardize its diagnostic suspicion [7]. This consensus statement turned out to be particularly accurate in clinical trials, with a positive predictive value of “typical” pattern of up to 87.8% in an epidemic setting [8].

Up to this writing, there are no studies to date comparing the CT findings of COVID-19 patients between the first and second waves. Therefore, the CT findings of hospitalized COVID-19 patients during both the first and second waves of the pandemic were analyzed, with the aim of verifying whether there were differences between the two groups and, above all, whether the experience acquired during the time elapsed between the two waves could have contributed to better management of the individuals infected.

## Methods

### Patient population and study design

This observational, retrospective, monocentric study was approved by our local institution review board (IRB) and informed consent was waived by the IRB due to its retrospective nature. All patients, both hospitalized and those who accessed to the Emergency Department, who underwent CT for suspected COVID-19-related pneumonia, from February 27 to March 27, 2020 and then from October 26 to November, 24 2020, which represented the peak periods of the first and second waves in Italy respectively, were consecutively enrolled.

In the case of suspected COVID-19 pneumonia, imaging workflow in our hospital consisted of a chest X-ray, eventually followed by a CT scan if radiography provided negative or uncertain findings. During the first wave, oro- and nasopharyngeal swabs were performed only in the case of close contacts with confirmed or probable COVID-19 patients and/or in case of suspicious radiological findings for COVID-19. During the second wave, according to its availability, RT-PCR was performed for nearly every patient admitted to the hospital as a protocol screening. The inclusion criteria were (1) symptomatic patients presenting fever (of unknown origin) or respiratory symptoms such as cough or dyspnea and (2) patients undergoing CT at our institute.

The exclusion criteria were (1) patients with suspected COVID-19-related interstitial pneumonia investigated only with other imaging techniques (X-ray or US), (2) patients with suspected COVID-19-related interstitial pneumonia with CT performed in different hospital, and (3) severe motion artifacts on chest CT (*n* = 46).

Overall, a total of 1247 CT scans were obtained (569 during the first wave and 678 during the second wave).

### Clinical data

The RT-PCR results were considered to be the reference standard and were extracted from electronic patient medical records of the hospital database. Since some patients had undergone more than one test, only RT-PCR performed within 24 h from the CT scan were considered.

RT-PCR was a 2-site test, performed using oro- and nasopharyngeal swabs; patients with RT-PCR not available or not carried out were excluded from the analysis (*n* = 171, 109 during the first wave and 62 during the second wave).

### CT acquisition technique

Chest CT acquisitions were obtained with the patients in supine position during end-inspiration without intra venous contrast medium injection. Expiration scan was not performed.

CT scans were performed on two CT scanners dedicated only to patients with suspected COVID-19:

1. 64-slice CT (GE Medical System, Light speed VCT 64 slice), with the following technical parameters: tube voltage: 120 kV; tube current modulation 100–250 mAs; spiral pitch factor: 0.98; collimation width: 64 × 0.625. Reconstructions were made with convolution kernel BONEPLUS at a slice thickness of 1.25 mm
2. 128 slice CT (PHILIPS Ingenuity CT 128 slice, with the following technical parameters: tube voltage: 120 kV; tube current modulation 100–250 mAs; spiral pitch factor: 1.224; collimation width: 64 × 0.625. Reconstructions were obtained with convolution kernel Y-SHARP at a slice thickness of 1 mm

After each patient chest CT examination, passive air exchange and a decontamination of CT room was performed with surface disinfection at 62–71% ethanol or 01% sodium hypochlorite.

### CT image analysis

All CT examinations were reviewed by accessing to the PACS (Picture Archiving and Communication System) of the hospital by 4 radiologists with more than 10 years of experience in thoracic imaging.

CT features were classified according to the four categories proposed by RSNA Expert Consensus (Table 1) [7].

**Table 1:** RSNA Chest CT Classification System for Reporting COVID-19 pneumonia [3]

COVID-19 pneumonia pattern	CT features
“Typical”	- Peripheral, bilateral, GGO with or without consolidation or visible intralobular lines (“crazy- paving”) - Multifocal GGO of rounded morphology with or without consolidation or visible intralobular lines (“crazy-paving”) - Reverse halo sign or other findings of organizing pneumonia
“Indeterminate”	The absence of typical imaging feature of COVID-19 pneumonia and the presence of: - Multifocal, diffuse, perihilar or unilateral GGO with or without consolidation lacking a specific distribution or features - Few very small GGO with a non-rounded and non-peripheral distribution
“Atypical”	The absence of typical or indeterminate features of COVID-19 pneumonia and the presence of: - Isolated lobar or segmental consolidation without GGO - Nodular pattern (centrilobular, “tree- in-bud”) - Cavitation - Smooth interlobular septal thickening with pleural effusion
“Negative”	No features suggesting pneumonia

**Fig. 1:**
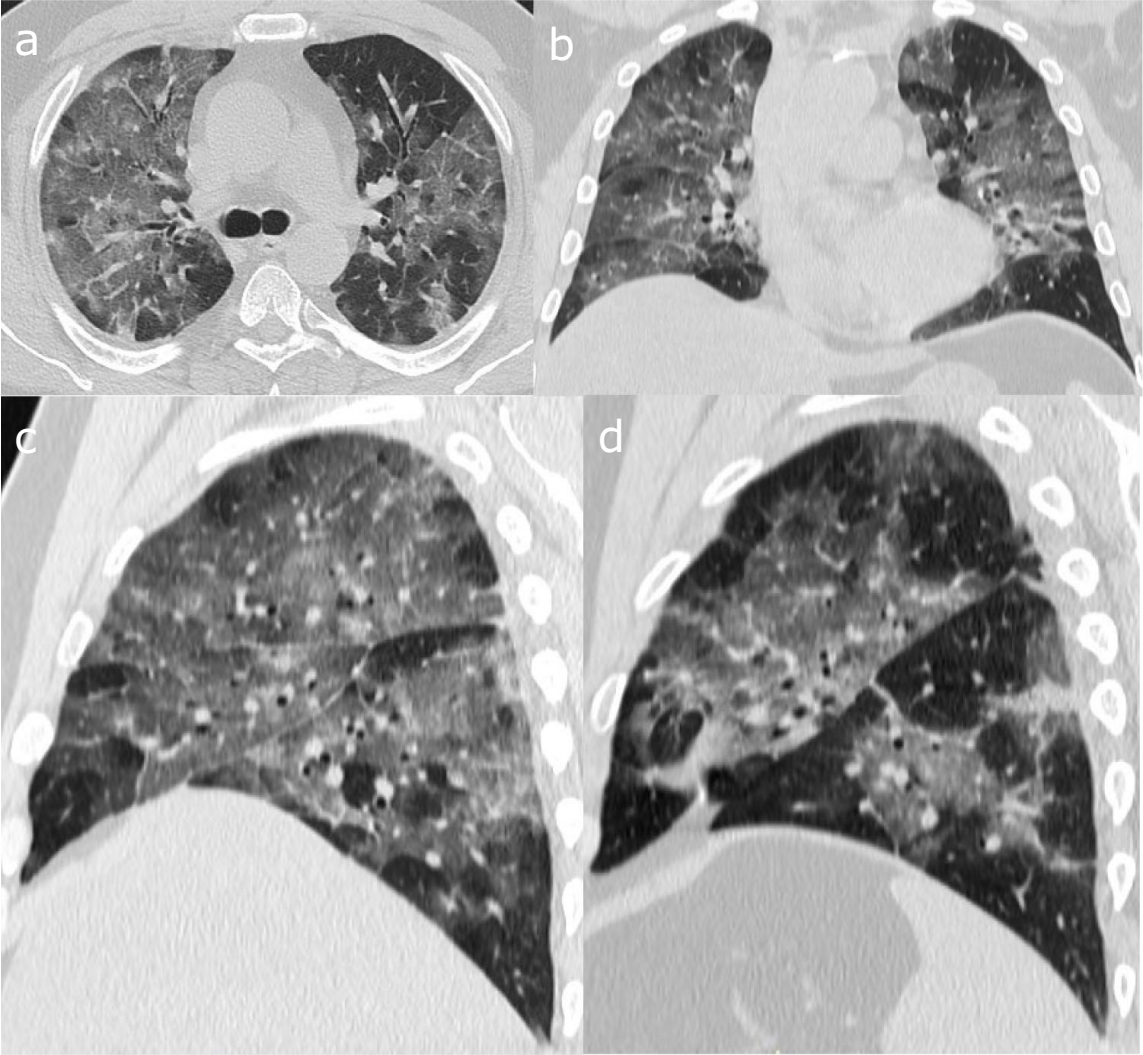
Visual score assessment. Diffuse bilateral ground-glass involving all five lobes (**a-d**). Multiplanar reconstructions allow a better evaluation of the visual score. Score of the right lung (**c**): right superior lobe = 4, middle lobe = 4, right inferior lobe = 3. Score of the left lung (**d**): left superior lobe = 3, left inferior lobe = 3. Total score: 17/25

**Fig. 2:**
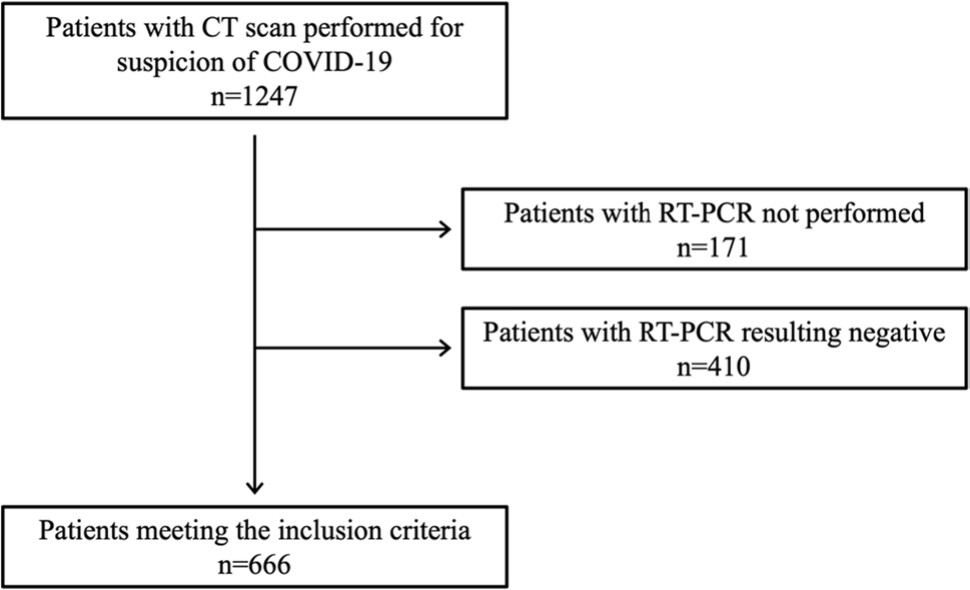
The workflow of patient data collection

To quantify the extent of disease, a semi-quantitative score was assigned to every patient with typical COVID-19 pattern on chest CT on the basis of the area involved and presenting with the findings of viral pneumonia, i.e., GGO, interlobular thickening, crazy-paving pattern, consolidation, reverse halo sign, and double reverse halo sign [9, 10]. Each of the five lung lobes was assessed for degree of involvement and scored as 0 (no involvement), 1 (< 5% involvement), 2 (5–25% involvement), 3 (26–49% involvement), 4 (50–75% involvement), or 5 (> 75% involvement), leading a total score ranging from 0 (no involvement) to 25 (maximum involvement) (Fig. 1). This system was an adaptation of a method previously used to describe idiopathic pulmonary fibrosis and has proven to correlate well with the degree of disease in pathologic specimens [11]. Patients with “typical” pattern but conspicuous motion artifacts, previous lobectomy or presence of atelectasis that did not allow severity score evaluation of the parenchyma were excluded from the analysis (*n* = 9, all during the second wave) (Fig. 2).

All data were anonymized and collected in a shared database.

### Statistical analysis

Data were expressed as means, standard deviation, ranges and frequencies. The Chi-squared, Fisher’s exact, ANOVA with Fisher’s least significant difference post hoc, and Mann–Whitney *U* tests were used. The Pearson correlation coefficient was calculated. All the tests were two-tailed. A *P*-value < 0.05 was considered statistically significant. All the statistical analyses were carried out using IBM SPSS 25.0 (SPSS Inc., Armonk, NY, USA).

## Results

During the first wave of COVID-19 (from February 27 to March 27, 2020), a total of 569 CT scans for suspected COVID-19-related pneumonia were obtained. However, only 211 patients had a positive RT-PCR result and were included in the study (211/569, 37.1%) while 249 were negative and 109 remained indeterminate since oro- and nasopharyngeal swabs were not collected. A diagnosis of COVID-19 infection was made in 211 patients (211/460, 45.9%), having a mean age of 64.52 years (range 21–94, SD 15.14); males were more frequently affected (144/211, 68.25%).

During the second wave of the COVID-19 pandemic (from 26 October to 24 November 2020), a total of 678 CT scans for suspected COVID-19-related pneumonia were obtained. However, only 455 patients had a positive RT-PCR result and were included in the study (455/678, 67.1%) while 161 were negative and 62 remained indeterminate since oro- and nasopharyngeal swabs were not performed. In particular, regarding the patients with positive results at RT-PCR, 198 patients (198/455, 43.5%) tested positive at the moment of their access to the Emergency Room while 257 (257/455, 56.5%) were known confirmed COVID-19 cases in self-isolation arriving at the Emergency Room realistically due to a worsening of their clinical picture. During the second wave, a diagnosis of COVID-19 infection was made in 455 patients (455/616, 73.9%), having a mean age of 68.26 years (range 12–100, SD 16.34); once again, males, were more frequently affected (283/455, 62.20%).

**Fig. 3:**
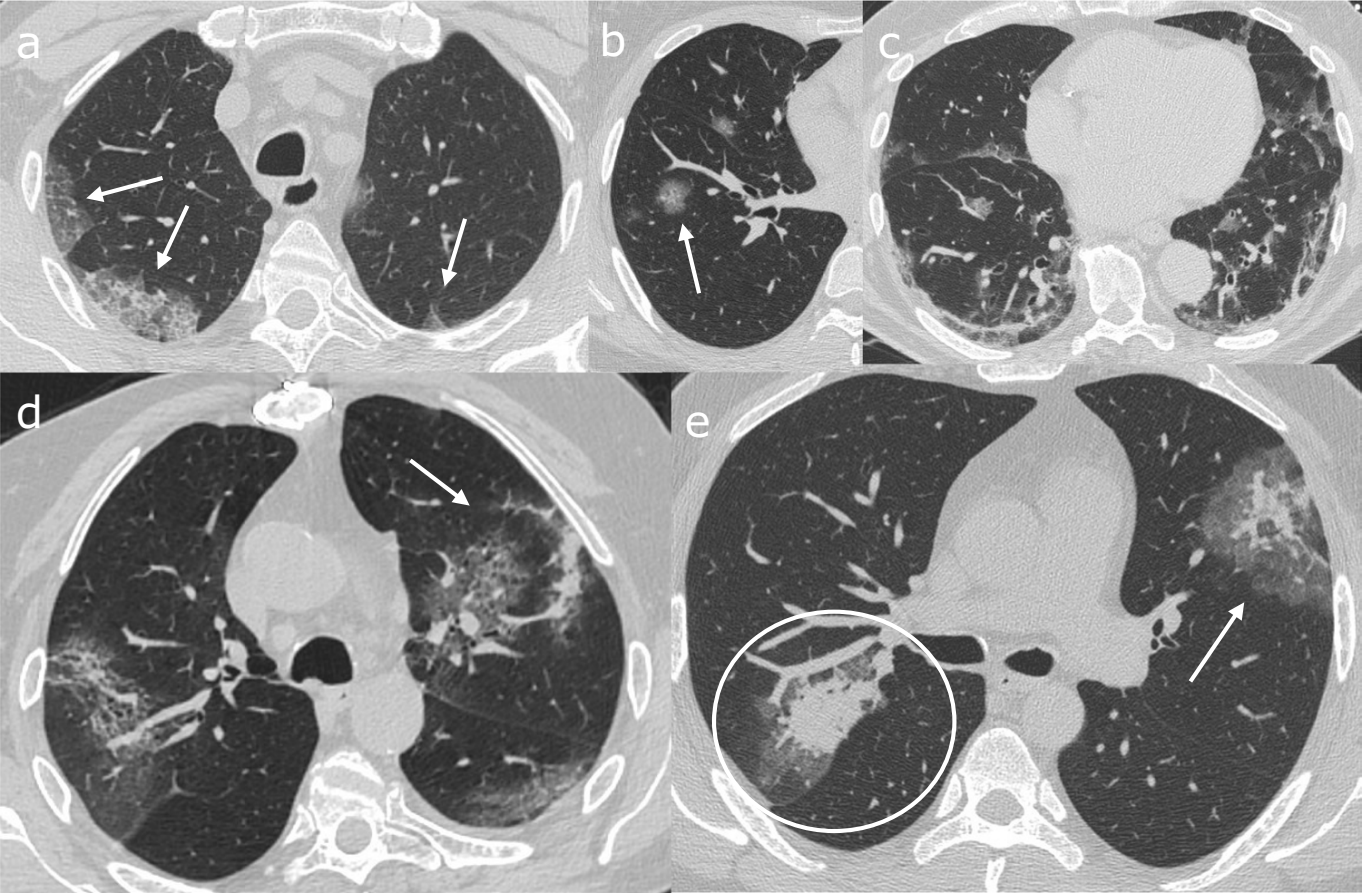
Radiological features of the “typical” pattern: **a** multiple, peripheral, subpleural ground-glass opacities associated with crazy-paving (arrows); **b** rounded ground-glass opacities (arrows); **c** organizing pneumonia characterized by multiple and patchy consolidations, with band-like pattern, peri-lobular opacities and ground-glass; **d** reverse halo sign (arrow); **e** halo sign (circle) and double reverse halo sign (also called target sign) (head arrow)

The analysis of the distribution of patterns documented an almost identical prevalence of patterns in both groups without statistically significant differences (*p* = 0.916) and the “typical” pattern was the most common pattern in both the first and the second waves (151/211, 71.6% vs. 315/455, 69.2%) (Fig. 3). The “indeterminate” pattern was seen in only a few cases (36/211, 17.1% vs. 83/455, 18.2%), followed by the “negative” pattern (17/211, 8.1% vs. 38/455, 8.4%) and the rarer “atypical” pattern (7/211, 3.3% vs. 19/455, 4.2%). No statistically significant differences were observed regarding CT pattern prevalence even between patients of the second wave with different indication for imaging (*p* = 0.621), i.e., patients who tested positive at the moment of their access to the Emergency Room (new COVID-19 diagnosis) and patients who had already been diagnosed with COVID-19 and arrived at the Emergency Room realistically due to a worsening of their clinical picture (already known COVID-19 infection) (Table 2).

**Table 2:** CT pattern prevalence in patients of the second wave who tested positive at the moment of their access to the Emergency Room (new COVID-19 diagnosis) and in patients who had already been diagnosed with COVID-19 and arrived at the Emergency Room realistically due to a worsening of their clinical picture (already known COVID-19 infection) (*p* < 0.05 indicates a significant difference)

Characteristics	New COVID-19 diagnosis	Known COVID-19 infection	*P* value
No. of COVID-19 patients during the second wave	198	257	
CT pattern prevalence			*p* = 0.621
“Typical”	138 (69.7%)	177 (68.87%)	
“Indeterminate”	39 (19.7%)	44 (17.12%)	
“Atypical”	8 (4.04%)	11 (4.28%)	
“Negative”	13 (6.57%)	25 (9.73%)	

The mean age of the patients with a “typical” pattern in both the first and the second waves was 66 years (SD 14.70), while the “atypical” and the “negative” patients presented a mean age of 69 (SD 18.49) and 63 years (SD 22.04), respectively; the patients with an “indeterminate” pattern showed a slightly higher mean age as compared to the other groups (72 years, SD 16.37), especially as compared to both the “typical” and the “negative” patients (*p* < 0.001).

According to the data, the mean semi-quantitative score during the first wave and the second wave of the COVID-19 pandemic was similar, 8.4 (SD 3.06) vs. 9.0 (SD 2.88) respectively, demonstrating however a significant difference (*p* = 0.042). Taking into account the data of both waves, a positive correlation between age and semi-quantitative score was found, with a statistically significant relationship existing between the two (*r* = 0.15, *p* = 0.001). No significant differences were noted between males and females regarding the CT score during either wave.

The main differences regarding the epidemiological and the radiological data between the first and the second waves are summarized in Table 3.

**Table 3:** Patient characteristics, CT pattern prevalence and semi-quantitative CT score in both waves (*p* < 0.05 indicates a significant difference)

Characteristics	First wave	Second wave	*P* value
No. of COVID-19 patients	211	455	
Gender			
Male	144 (68.25%)	283 (62.20%)	
Female	67 (31.75%)	172 (37.80%)	
Age (years)			
Mean ± SD	64.52 ± 15.14	68.26 ± 16.34	*p* = 0.005
CT pattern prevalence			*p* = 0.916
“Typical”	151 (71.6%)	315 (69.2%)	
“Indeterminate”	36 (17.1%)	83 (18.2%)	
“Atypical”	7 (3.3%)	19 (4.2%)	
“Negative”	17 (8.1%)	38 (8.4%)	
CT score of “Typical” patients			
Mean ± SD	8.4 ± 3.06	9.0 ± 2.88	*p* = 0.042

## Discussion

Since the COVID-19 outbreak, clinicians have shared their experiences in managing patients with significant diagnosis and treatment changes over time, as confirmed by the transition from the administration primarily of antivirals during the early phase to an increased use of steroids in the second phase [4].

Considering the present large case series, the fact that there was no statistically significant difference regarding pattern prevalence between the two waves demonstrated both the validity of the RSNA consensus [7] and the results of prior published studies [8]. In particular, as previously reported [12], COVID-19 tends to present itself with “typical” radiological features in the majority of cases.

Analyzing the mean ages for each type of COVID-19 CT pattern, patients with the “typical” pattern were found to be the younger patients (63 years). On the contrary, the fact that the older patients were those who most frequently presented non-typical COVID-19 features, especially the “indeterminate” pattern (72 years), had important implications since the consequent uncertainty of the radiological diagnosis could cause difficulties in their management.

Although several studies [13] have documented gender differences in COVID-19 patients, despite a higher male prevalence, no significant differences in disease severity expressed as a semi-quantitative CT score was observed.

Given the progressively increased availability of RT-PCR, it was interesting to note that CT changed from a primarily diagnostic to a prognostic role during the pandemic. This observation was in accordance with the evidence that many of the patients of the second wave already knew they were infected by COVID-19 and were admitted to the hospital following clinical worsening. In this perspective, evaluation of the CT score became essential for proper patient management, proving to be essential for deciding whether or not to hospitalize the patient in healthcare settings with limited resources and with a shortage of intensive care beds. However, although indications for CT have changed over time, radiological features of COVID-19 pneumonia remained unchanged.

The average age of the infected population is now believed to be lower compared to the pandemic outbreak in March 2020, possibly due to wider RT-PCR availability and the consequent reduction in missed diagnoses, especially in mild and asymptomatic young patients [3, 14]. In the present study, however, COVID-19 patients during the second wave of the pandemic were slightly but significantly older than those infected during the first wave (68.26 vs. 64.52, *p* = 0.005).

The semi-quantitative score used in the present study has already proven to be predictive of patient outcome, in terms of both need for hospitalization or ICU and mortality [15, 16]. In fact, even if this visual evaluation is more simplistic than radiomic software, it is still a reliable prognostic tool and allows clinicians a faster stratification of patients [17]. The present semi-quantitative analysis of extension of COVID-19 CT features showed similar score severity values during the two waves (8.4 vs. 9.0) but the patients who were admitted during the second wave presented a slightly greater, but significant, CT extension of viral pneumonia. Even if this small difference is of little to no clinical relevance, it can be speculated that the prognosis of COVID-19 patients during the second wave was slightly worse, which would justify the still elevated number of deaths from COVID-19 despite the better level of treatment achieved. This observation could be supported by the evidence of an older age of the patients admitted during the second wave, possibly implying that the infected patients who still required hospitalization during the second wave were the elderly and thus the most susceptible to viral infections [18] while the younger patients could be sufficiently managed outside hospitals. However, these hypotheses are merely speculative and further studies are needed to address them thoroughly.

The strengths of the present study came from its large sample size and its monocentric design, implying that a similar method during both waves made the comparison relevant; moreover, the number of studies describing more than one wave of the COVID-19 pandemic is still very limited. However, the present analysis has several limitations, including its retrospective nature, the reported RT-PCR limitations [19] and the fact that some clinical co-variants that may affect COVID-19 disease severity, such as patient BMI, his immunological status and the duration of symptoms prior to CT scanning were not taking into account. Moreover, since the study design considered only the radiological features of COVID-19 pneumonia at baseline, the evolution and changes over time of the disease imaging features have not been collected, as well as the time of onset of the disease. Finally, the generalizability of the present findings might be limited since only data on Italian patients were collected, although they still provide a picture of the situation in one country.

## Conclusions

The present study demonstrated that there was no statistically significant difference regarding CT pattern prevalence between the first and the second waves in Italy. Moreover, this study confirmed the validity of the RSNA consensus regarding COVID-19 pattern classification and the most frequent radiological COVID-19 features. Finally, non-typical COVID-19 features were more frequently observed in older patients and, therefore, should not be underestimated in the elderly population.

## Data Availability

The datasets used and/or analyzed during the current study are available from the corresponding author on reasonable request.

## Abbreviations

COVID-19: Coronavirus disease 2019
GGO: Ground-glass opacity
RSNA: Radiological Society of North America
RT-PCR: Reverse-transcription polymerase chain reaction
